# Hypnotism in Public Forbidden

**Published:** 1891-06

**Authors:** 


### Hypnotism in Public Forbidden.

The first official suppression of a public hypnotic entertainment in this country occurred in Cincinnati, where the Health Commissioner, Dr. J. W. Prendegrast, advised the licensing authorities of that city that hypnotic exhibitions should be forbidden.

Dr. Prendergast takes the ground that the indiscriminate application of hypnotism is in a large proportion of cases injurious to the mental health of the subjects. The Common Council has interdicted these exhibitions, and adopted an ordinance making them a misdemeanor.

